# Impact of conflict on maternal and child health service delivery – how and how not: a country case study of conflict affected areas of Pakistan

**DOI:** 10.1186/s13031-020-00271-3

**Published:** 2020-05-27

**Authors:** Jai K. Das, Zahra Ali Padhani, Sultana Jabeen, Arjumand Rizvi, Uzair Ansari, Malika Fatima, Ghulam Akbar, Wardah Ahmed, Zulfiqar A. Bhutta

**Affiliations:** 1 Division of Women and Child Health, Aga Khan University, Karachi, Pakistan; 2 Department of Paediatrics and Child Health, Aga Khan University, Karachi, Pakistan; 3 Center of Excellence in Women and Child Health, Aga Khan University, Karachi, Pakistan; 4 Centre for Global Child Health, The Hospital for Sick Children, 686 Bay Street, Toronto, ON M5G 0A4 Canada

**Keywords:** Conflict, Humanitarian, Balochistan, FATA, Pakistan, Maternal health, Child health, Nutrition

## Abstract

**Introduction:**

In conflict affected countries, healthcare delivery remains a huge concern. Pakistan is one country engulfed with conflict spanning various areas and time spans. We aimed to explore the effect of conflict on provision of reproductive, maternal, newborn, child and adolescent health and nutrition (RMNCAH&N) services and describe the contextual factors influencing the prioritization and implementation in conflict affected areas of Pakistan (Balochistan and FATA).

**Method:**

We conducted a secondary quantitative and a primary qualitative analysis. For the quantitative analysis, we stratified the various districts/agencies of Balochistan and FATA into the conflict categories of minimal-, moderate- and severe based on accessibility to health services through a Delphi methodology with local stakeholders and implementing agencies and also based on battle-related deaths (BRD) information from Uppsala Conflict Data Program (UCDP). The coverage of RMNCAH&N indicators across the continuum of care were extracted from the demographic and health surveys (DHS) and district health information system (DHIS). We conducted a stratified descriptive analysis and multivariate analysis using STATA version 15. The qualitative data was captured by conducting key informant interviews of stakeholders working in government, NGOs, UN agencies and academia. All the interviews were audiotaped which were transcribed, translated, coded and analyzed on Nvivo software version 10.

**Results:**

The comparison of the various districts based on the severity of conflict through Delphi process showed that the mean coverage of various RMNCAH&N indicators in Balochistan were significantly lower in severe- conflict districts when compared to minimal conflict districts, while there was no significant difference between moderate and severe conflict areas. There was no reliable quantitative data available for FATA. Key factors identified through qualitative analysis, which affected the prioritization and delivery of services included planning at the central level, lack of coordination amongst various hierarchies of the government and various stakeholders. Other factors included unavailability of health workforce especially female workers, poor quality of healthcare services, poor data keeping and monitoring, lack of funds and inconsistent supplies. Women and child health is set at a high priority but capacity gap at service delivery, resilience from health workers, insecurity and poor infrastructure severely hampers the delivery of quality healthcare services.

**Conclusion:**

Conflict has severely hampered the delivery of health services and a wholesome effort is desired involving coordination amongst various stakeholders. The multiple barriers in conflict contexts cannot be fully mitigated, but efforts should be made to negate these as much as possible with good governance, planning, efficiency and transparency in utilization of available resources.

## Background

Pakistan is the sixth most populous country (207 million) in the world with 64% of its population living in rural areas [1, 2]. It has a diverse and a vast landscape of 770,875 km^2^ comprising of four provinces – Balochistan, Khyber Pakhtunkhwa (KP), Punjab and Sindh and four federal territories – Azad Kashmir, Gilgit Baltistan, Federally Administered Tribal Areas (FATA) and Islamabad [3], with FATA being recently merged with KP.

**Fig. 1:**
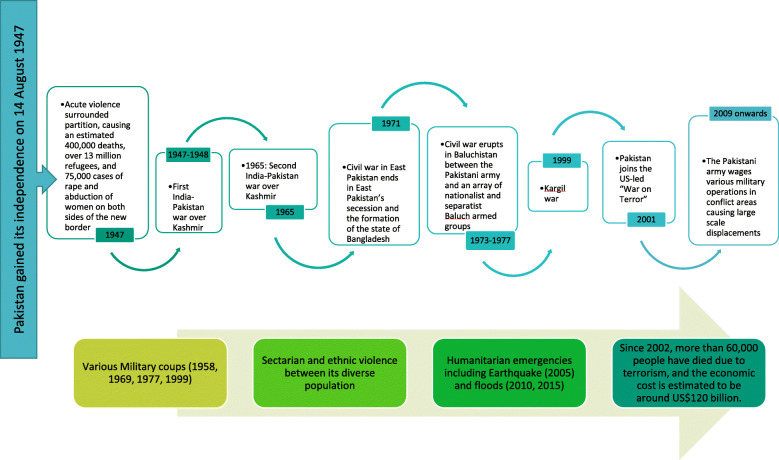
Conflict timeline of Pakistan

Pakistan is a volatile geopolitical region which has faced various inter-state and intra-state conflicts since its independence in the year 1947 (Fig. 1). Pakistan’s conflict and political instability over the decades has been in large measure due to the struggle between major actors— the civilian wing of the state, the military, the Islamists and the relations with bordering and international countries. It started at the partition from British India in 1947, and led to subsequent wars with India. Regional developments, such as the Kashmir dispute with India, further partitioning of the state in 1971, the wars in Afghanistan, and the recent U.S.-led war on terror, have affected Pakistan’s internal dynamics. Internal developments which have threatened state’s authority include the Islamic establishment, sectarian and ethnic violence between its diverse populations. There have been various conflicts between the inhabitants of the Balochistan province and the government of Pakistan with the goal of autonomy. And with the Taliban’s increasing power in Afghanistan during the 1990s and the war on terror in 2001, Pakistan’s tribal areas (FATA) have witnessed domestic conflicts which escalated after the international drone attacks. Terrorism and the ‘war on terror’ have been costly for Pakistan – both in terms of human loss and economic costs and since 2002, terrorism has killed more than 50,000 people, which has an estimated economic cost of around US$120 billion [4].

Pakistan failed to achieve most Millennium Development Goals (MDGs) including MDG 4 and 5 and there is still a high burden of maternal and child morbidity and mortality and undernutrion. Pakistan has been ranked as the second lowest progressing country in south Asia according to the Sustainable Development Goals (SDGs) index [5]. Pakistan is striving hard to eradicate poverty and hunger, as 38% [6] of the population is living below poverty line although the poverty headcount ratio has decreased from 6.1% in 2013 to 4% in 2015 [7]. Fragile and Conflict States (FCS) reports decrease in under five mortality from 147 per 1000 live births in year 1990 to 78 in year 2015 [8]. Similarly, Pakistan Demographic and Health Survey (PDHS) shows that infant mortality has reduced in the year 2012 from 74 per 1000 live births to 62 per 1000 live births and under 5 mortality has reduced from 89 to 75 per 1000 live births in year 2017. There have been improvements in coverage of essential on reproductive, maternal, newborn, child and adolescent health and nutrition (RMNCAH&N) including vaccination coverage but the progress is slower and uneven [9].

**Fig. 2:**
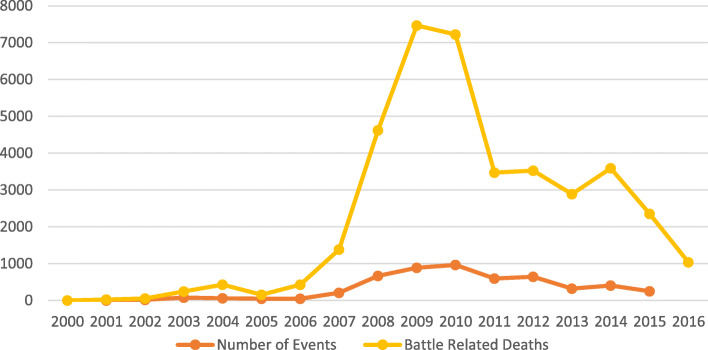
Conflict related events and battle related deaths in Pakistan

**Fig. 3:**
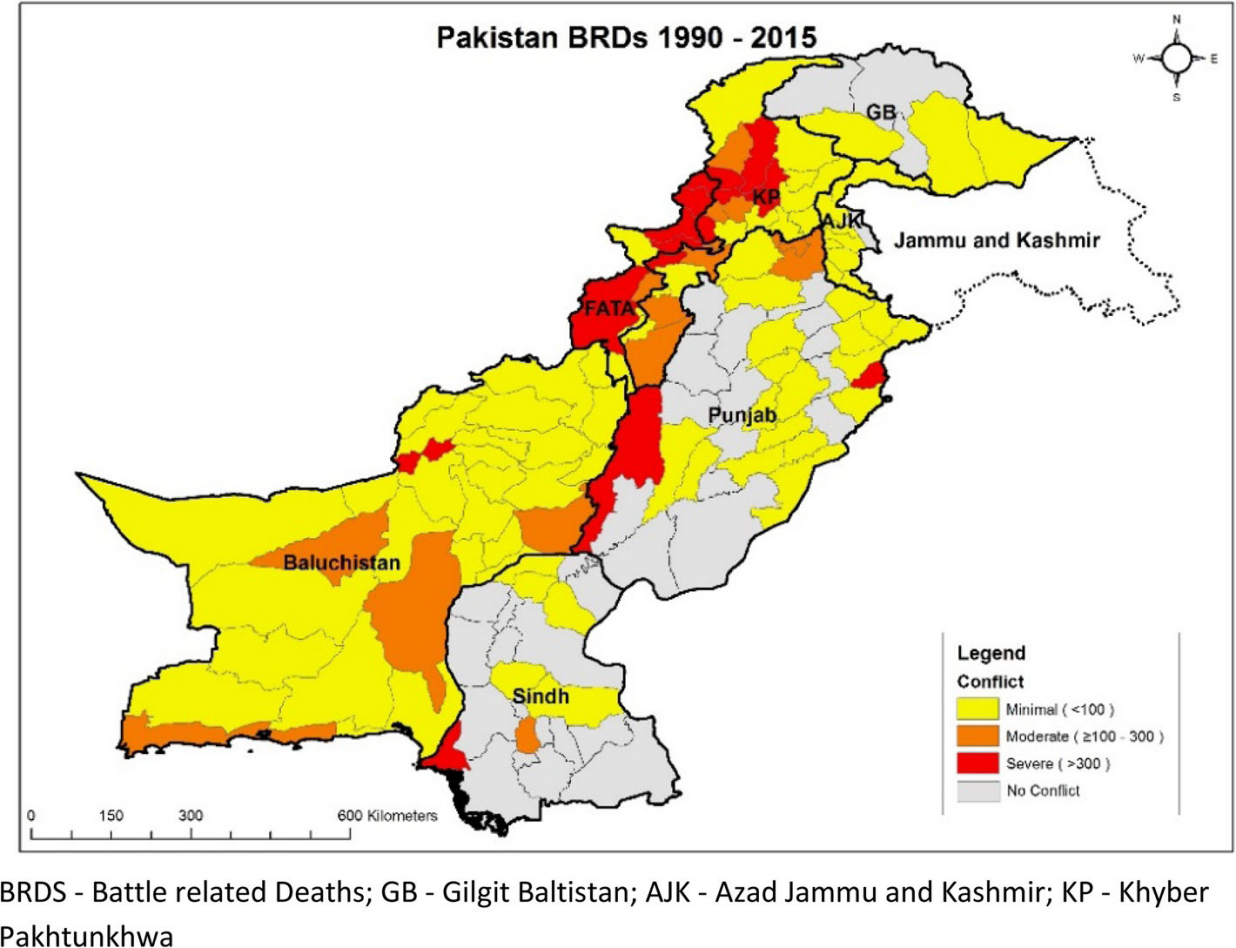
Geographic distribution of Battle related deaths in Pakistan

There should be specific policies and strategies to deliver health services in conflict areas but these are mostly missing and hence this study presented here is part of a multi-country study coordinated by the Bridging Research and Action in Conflict Settings for the Health of Women and. Children (BRANCH) Consortium focusing on ten conflict-affected countries: Afghanistan, Colombia, DRC, Mali, Nigeria, Pakistan, Somalia, South Sudan, Syria, Yemen [10]. This country case study focuses on the recent conflict in areas of Pakistan which started after the year 2001, and escalated after 2006 as represented by the Battle Related Deaths (BRDs) and incidents according to Uppsala Conflict Data Program (UCDP) [11] (Fig. 2). This study focuses on two areas of Pakistan – Balochistan with a specific focus on the Makran belt which is southern region of Balochistan and includes the districts of Gwadar, Keich and Panjgur and FATA due to the chronic nature of conflict in these areas (Table 1, Fig. 3, Table 2). The aim of this study is to explore the provision of RMNCH&N interventions in Balochistan and FATA and describe the factors that influence the decision making and implementation.

**Table 1:** Geographic Focus and History of Conflict

The geographic focus of this study is on the province of Balochistan and the tribal areas of FATA. Balochistan is the largest province of Pakistan which covers an area of 347,190 km^2^ [12] with a population of 12,344,408 people [1]. Balochistan comprises of 26 districts where Balochs and Pashtuns are the major ethic groups. Besides the historical and political reasons, the social factors such as ethnicity and religion have also played a vital role in the continuance of the conflict. Lack of representation at the decision-making level and low quotas for political representation are the prominent factors that have added more misery to the ongoing problems. The history of the conflict in the province goes back to the independence of Pakistan in 1947. The first conflict arose in 1948, when Kalat, a part of the current Balochistan, chose independence. This was followed by further conflict in 1958–59 and 1963–69 and these conflicts were about the One Unit policy and military bases in Balochistan. In 1973–79, the provincial government of Balochistan was dismissed, which led to an armed insurgency and revolt. From 2005 to now, there have been several political and ethnic issues driving the protracted and ongoing conflict between the inhabitants of the Balochistan province and the government of Pakistan with the goal of autonomy or possibly independence.	
FATA with a population of 5,001,676 people [1], comprises an area of 27,224 sq. km [13] of rugged terrain. FATA is characterized by very rich ethnic diversity, cultural heritage and strong tribal structure. FATA comprises of seven tribal agencies and six frontier regions. FATA have generally been calm and peaceful throughout Pakistan’s history but peace was ruined, as fallout of War on Terror and subsequent military operations since 2003–04. FATA people have their own “customary law” composed of traditional legal codes and legalistic, institutionalized procedure which is abided by its subjects regardless of their status and social position [14]. But with the arrival of Al-Qaeda and other foreign terrorists after the US attack on Afghanistan in 2001, the actual representation of Pashtuns was hijacked and whoever among the tribesmen that rose against the terrorists and extremists was shot dead by the militants and local leaders of the Pashtun tribes are deliberately targeted. However, with increased resistance in Afghanistan, the tribal areas have been gradually transformed into a kind of war zone. The Pakistani military launched multiple operations in FATA. In May 2018, FATA was dissolved and became part of the KP province, and the jurisdiction of the high courts is now extended to the tribes. This was part of a counterterrorism strategy, since the lack of development and access to education, infrastructure and justice has long been pointed out as something that eases the recruitment to militant movements.

**Table 2:** Demographic characteristics of selected study sites

	Balochistan	FATA
		
**Population**		
***Area***	347,190 sq. km.	27,224 sq. km
***Population***^a^	12,344,408	5,001,676
***Battle Related Deaths 2000–2017***	2698	23,575
***Number of Healthcare Facilities***	668^b^	201^c^
**Health Service Coverage**^**d**^		
***Use of any modern method of family planning***	14.0	13.7
***% of demand satisfied***	47.9	66.4
***Total Fertility Rate (Births per woman)***	4.0	4.8
***Pregnant women receiving iron folate***	17.1	50
***Last birth protected by Neonatal tetanus***	26.7	38.5
***Skilled birth attendance***	38.2	52.1
***Institutional delivery rate***	34.6	49.1
***PNC within 2 days (mother/newborn)***	37.9	31.8
***BCG***	46.6	54.9
***Three doses of polio vaccine***	57.1	82.5
***Three doses of*** DPT-HepB-Hib	40.1	46.7
***Measles immunization coverage***	33.3	34.5
***Full vaccination coverage***	28.8	30.4
***Number of children with symptoms of ARI***	58	32
***Child acute respiratory infection care seeking***	62.2	70.6
***Child fever care seeking***	60	62.9
***Children with diarrhea***	95	48
***Children with diarrhea treated with ORS***	51	23.9

**Key: a:** Pakistan census 2017; **b:** Health Facility Assessment – Balochistan Provincial Report 2012**; c:** Health Facility Assessment – FATA Provincial Report 2012; **d:** Pakistan Demographic Survey 2017–2018

*PNC* Post natal care, *BCG* Bacille Calmette-Guérin, *ARI* Acute respiratory infection), *ORS* Oral Rehydration Solution

## Methods

We conducted a quantitative analysis of secondary data and a primary qualitative analysis. The quantitative component focused on estimating the coverage, trends and the impact of conflict on RMNCAH&N services and the primary qualitative component aimed to understand the underlying contextual factors that influence the decision making and implementation of RMNCAH&N services.

### Quantitative methodology

The following sources were used to obtain data for the quantitative analysis

- Conflict – Conflict data was extracted from the UCDP [11] including date, location, type and number of events, and fatalities
- Health – Coverage data on key RMNCAH&N coverage indicators was extracted from nationwide household surveys: PDHS (2006 and 2012–13) [9, 15]
- Health Facility Data - The District Health Information System (DHIS) which uses the DHIS to collect and manage data from the health facilities in FATA and Balochistan (2011–2018)

We stratified the districts of Balochistan and agencies of FATA, on the severity of conflict which was based on two classification methodologies. First was based on number of BRDs from the UCDP (Minimal – less than 100 BRDs, Moderate – 100- 300 BRDs, Severe - greater than 300 BRD) and for the second we conducted a Delphi exercise. For the Delphi exercise, we employed a purposive sampling strategy, disseminating the survey through key stakeholders. The expert panel comprised of thirty nine key stakeholders from government, UN agencies, NGOs and academia at the central, provincial and district level. These stakeholders were provided with a rating questionnaire over email and each of the experts rated the districts/agencies as mild-, moderate- and severe- conflict based on accessibility for the health-care providers and the community to seek care during the conflict period (Likert Scale) for the period 2006–2015. Non-responders received reminder over emails, and were excluded after two reminders. The votes of all panel members are weighed equally within the Delphi process and districts in Balochistan and agencies in FATA were then grouped on the basis of severity of conflict.

The coverage of RMNCAH&N indicators were extracted from the PDHS 2006 and 2012 (contraceptive use, antenatal care (ANC), skilled birth attendance (SBAs), facility birth, immunization (BCG, DPT3/Penta3), exclusive breastfeeding, continued breastfeeding, vitamin A supplementation, care-seeking for acute respiratory infections (ARI), use of oral rehydration therapy) and facility data from DHIS (OPD visits, ANC, post-natal care (PNC), livebirths, stillbirths, low birth weight, vaccum/forceps deliveries, ARI, pneumonia, diarrhea, DPT3/Penta3, measles, tetanus toxoid). The Composite Coverage Index (CCI) [16] was calculated as a weighted coverage mean of eight essential interventions that represent broad categories of the continuum of care. The four categories are family planning, maternal and newborn care, immunization, and case management of sick children. Each continuum stage was given equal weight and the CCI was then calculated as below.

$$ CCI=\raisebox{1ex}{$1$}\!\left/ \!\raisebox{-1ex}{$4$}\right.\left( FPS+\frac{SBA+ ANCS}{2}+\frac{2 DPT3+ MSL+ BCG}{4}+\frac{ORT+ CPNM}{2}\right) $$

(Note that FPS indicates family planning needs satisfied (related to contraceptive use) and CPNM refers to care seeking for ARI)

For the DHS data; means and mean differences of the net coverage estimates of the various indicators were calculated between the years 2006–2012 and compared across conflict subgroups using the Student’s t-tests and one-way analysis of variance methods. Post-hoc comparisons were conducted with the Tukey’s multiple comparison methods. We then examined the impact of conflict on coverage indicators using binary logistic regression approach. The primary exposure was conflict status and the effect exposure was accessed adjusting for time, place of residence, maternal education and wealth status. In addition, the analysis was adjusted for survey design and sampling weights through ‘svy’ routine in STATA version 15 [17]. Bivariate analysis was conducted to evaluate the independent effect of primary exposures on outcomes and the multivariate adjusted models were developed with time with and without covariates to assess the impact of these exposures with time on the outcome. The results reported as odds ratios (OR) with 95% confidence intervals and the *p*-value less than 0.05 was considered significant.

### Qualitative methodology

We undertook a primary qualitative data collection through key informant interviews which included stakeholders from Government, development partners, NGOs and academia to capture varied opinions. For this purpose, we selected stakeholders with experiences in implementation, policy making, and in monitoring and evaluation. Participants were identified through purposive sampling and snowball technique. A semi-structured interview guide was developed and tested which focused on coordination, finances, governance, intervention prioritization, security, monitoring and evaluation, health workforce and service delivery. Interviews lasted approximately for an hour and were conducted in Urdu which were audio recorded and then transcribed and translated in English by a bi-lingual translator. Latent content analysis was used to analyze the data and thematic analysis (combining an inductive and deductive approach) was used with the assistance of NVivo version 10.0 software [18].

## Results

The quantitative analysis shows the impact and trends of conflict and the qualitative component focuses on the process of decision making, enablers/barriers, prioritization and implementation of RMNCAH&N services in Balochistan and FATA.

### Quantitative

We observed DHIS data from the year 2011 to 2018; and more than half of the data was missing from both Balochistan and FATA and for some specific indicators the range of missing data ballooned to over 90%. The available data had some serious quality issues, hence, with this high level of attrition and unreliability, we could not attempt any reliable analysis.

We were also unable to perform analysis for FATA due to unavailability of quantitative data from the household surveys that were conducted in Pakistan prior to 2017. For Balochistan; we had district specific data from PDHS for the year 2006 and 2012, these years resonate well with our objectives as after 2006, the conflict escalated and reached the peak in the year 2011, hence the comparisons adequately shows the impact of conflict on the coverage of various RMNCAH&N indicators. The comparison of the various districts classified on the severity of conflict according to BRD showed that there was no significant difference in the mean coverage of selected indicators between the various conflict districts but in fact the mean coverage was higher in conflict districts for both the DHS 2006 and DHS 2013 estimates.

**Fig. 4:**
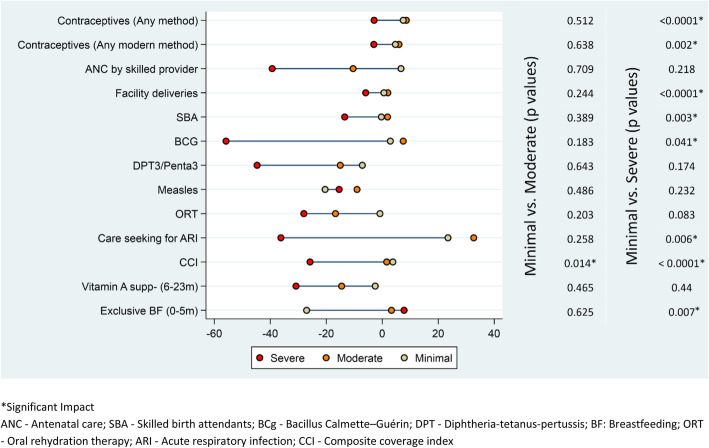
Mean Difference in the intervention coverage of Balochistan by conflict status from 2006 to 2012

The analysis based on the district grouping of conflict severity through Delphi methodology showed that the mean coverage of indicators was comparable in the year 2006 (pre-conflict) between the districts grouped as minimal, moderate or severe conflict. The bivariate analysis showed that the mean difference in the coverage of indicators for the period 2006–2012 was significantly lower for severe conflict when compared to minimal conflict provinces for a range of indicators (Fig. 4). The multivariate analysis shows that the mean difference in coverage for indicators including contraceptive-any method (OR: 0.51, 95% CI: 0.35–0.76), contraceptive – any modern method (OR: 0.63, 95% CI: 0.43–0.93), facility delivery (OR: 0.28, 95% CI: 0.16–0.48), SBA (OR: 0.42, 95% CI: 0.25–0.72), exclusive breast feeding (OR: 0.28, 95% CI: 0.1–0.77), BCG (OR: 0.38, 95% CI: 0.15–0.94), care-seeking for ARI (OR: 0.28, 95% CI: 0.10–0.83), was significantly lower for severe conflict districts when compared to minimal conflict districts, while there was no significant difference in the coverage for various indicators between the minimal and moderate severe groups (Table 3).

**Table 3:** Multivariate analysis for Balochistan for the years 2006–2012

Outcomes	Conflict Type	Bivariate				Adjusted			
		***OR***	***95% CI***		***p-value***	***OR***	***95% CI***		***p-value***
**Contraceptives (Any method)**	Minimal	Ref.				Ref.			
	Moderate	0.80	0.42	1.55	0.512	0.88	0.48	1.62	0.686
	Severe	0.42	0.28	0.63	< 0.0001	0.51	0.35	0.76	0.001
**Contraceptives (Any modern method)**	Minimal	Ref.				Ref.			
	Moderate	0.86	0.45	1.63	0.638	0.95	0.52	1.72	0.854
	Severe	0.51	0.34	0.78	0.002	0.63	0.43	0.93	0.019
**ANC by skilled provider**	Minimal	Ref.				Ref.			
	Moderate	1.10	0.67	1.81	0.709	1.46	0.93	2.31	0.100
	Severe	0.66	0.35	1.28	0.218	0.92	0.57	1.49	0.740
**Facility Deliveries**	Minimal	Ref.				Ref.			
	Moderate	1.33	0.82	2.14	0.244	1.70	0.99	2.92	0.054
	Severe	0.23	0.11	0.46	< 0.0001	0.28	0.16	0.48	< 0.0001
**Skilled Birth Attendant**	Minimal	Ref.				Ref.			
	Moderate	1.24	0.76	2.01	0.389	1.59	0.91	2.78	0.101
	Severe	0.33	0.16	0.68	0.003	0.42	0.25	0.72	0.001
**BCG**	Minimal	Ref.				Ref.			
	Moderate	1.89	0.74	4.85	0.183	2.33	0.86	6.3	0.094
	Severe	0.34	0.12	0.96	0.041	0.38	0.15	0.94	0.036
**DPT/Penta 3**	Minimal	Ref.				Ref.			
	Moderate	0.84	0.41	1.74	0.643	0.96	0.47	1.97	0.917
	Severe	0.48	0.17	1.39	0.174	0.54	0.21	1.4	0.200
**Measles**	Minimal	Ref.				Ref.			
	Moderate	1.41	0.53	3.73	0.486	1.61	0.64	4.06	0.311
	Severe	1.76	0.70	4.44	0.232	2.01	0.74	5.46	0.169
**Care Seeking for ARI**	Minimal	Ref.				Ref.			
	Moderate	0.54	0.19	1.58	0.258	0.71	0.25	2.05	0.524
	Severe	0.22	0.08	0.64	0.006	0.28	0.1	0.83	0.022
**ORT**	Minimal	Ref.				Ref.			
	Moderate	0.61	0.28	1.31	0.203	0.75	0.38	1.46	0.395
	Severe	0.55	0.28	1.08	0.083	0.64	0.31	1.31	0.223
**Vitamin A (6–23 months)**	Minimal	Ref.				Ref.			
	Moderate	0.79	0.41	1.51	0.465	0.81	0.44	1.5	0.501
	Severe	0.81	0.47	1.39	0.440	0.86	0.51	1.46	0.578
**Exclusive Breast feeding (0-5 months)**	Minimal	Ref.				Ref.			
	Moderate	0.81	0.35	1.88	0.625	0.96	0.42	2.21	0.918
	Severe	0.27	0.11	0.70	0.007	0.28	0.1	0.77	0.015

### Qualitative

**Fig. 5:**
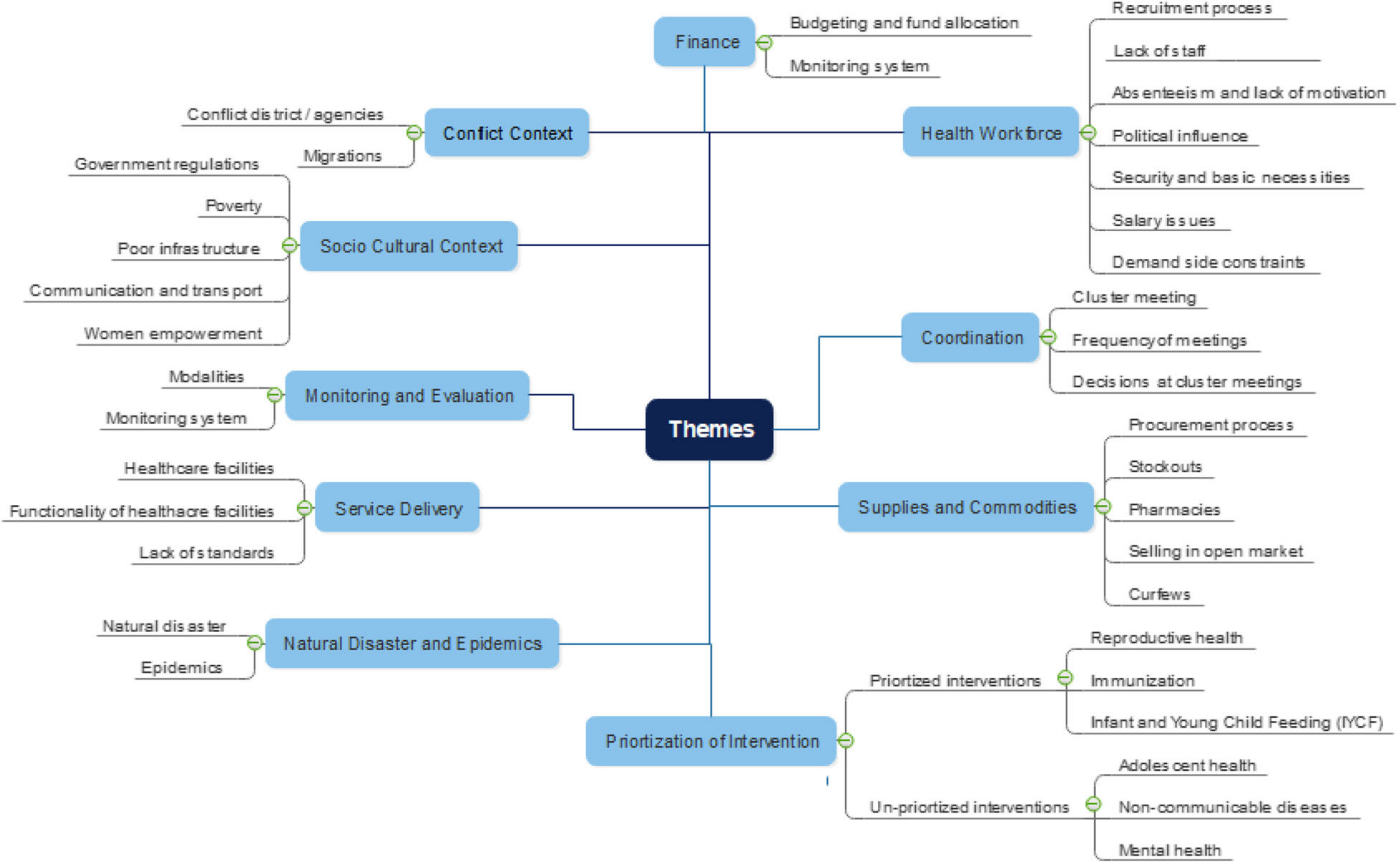
Themes and Categories from Key Informant Interviews

A total of 39 stakeholders were interviewed and the participant demographics are shown in Table 4. Below we detail the major themes and summarize the opinions as reported by the majority of the key informant interviews (Fig. 5).

**Table 4:** Participant Demographics of Key Informant Interviews

Variable	Key Informant Interviews	
	Number	(%)
**Sample size**		
Number of participants	39	
**Location**		
Balochistan	18	(46.1)
FATA	18	(46.1)
Islamabad	03	(7.6)
**Sex**		
Male	35	(89.7)
Female	04	(10.2)
**Age, years**		
20–30	05	(12.8)
31–40	09	(23)
41–60	23	(58.9)
Missing Values	02	(5.1)
**Education**		
Secondary Education	00	(00)
Graduate	12	(30.7)
Masters or Other Advanced Degree	27	(69.2)
**Experience, years**		
Below 10	07	(17.9)
10–20	15	(38.4)
21–30	12	(30.7)
30 Above	04	(10.2)
Missing Values	01	(2.5)
**Organization**		
Development Partners	03	(7.6)
Government	26	(66.6)
NGOs	08	(20.5)
Academia	02	(5.1)
**Type of staff**		
Center Based	15	(38.4)
District Based	14	(35.8)
Facility Based	08	(20.5)
Academia	02	(5.1)

*FATA* Federally Administered Tribal Areas, *NGOs* Non- governmental Organizations

#### Conflict context

Since independence, FATA and Balochistan have been the neglected part of the country and in the midst of various conflicts due to political, social, religious and sectarian reasons. In FATA – the agencies of North/South Waziristan, Khurram and Khyber agency and some areas of Orakzai, Bajaur and Mahmond were rated as most severely affected by conflict and in Balochistan- Makran division was identified as the most affected particularly the district of Keich and parts of Panjgur and some of the areas in FATA and Makran division are even marked as no-go areas (red zone). Security agencies have conducted various operations and most notably the operation Zarb-e-Azb which had led to a marked improvement in the security situation. These insurgency operations in FATA and Makran division had forced many people to move to the adjoining districts for their safety (internally displaced persons (IDPs)) and initially lived in camps provided by the government but later settled in those particular districts. But after the operation, most of the people have returned back (returnees), although there were major infrastructure issues. While, recently the security has improved in many of these areas but there are still pockets under conflict.

*“The situation has improved with time, but there are still some ‘no go’ areas like in South Waziristan and this improvement is due to military operations like ‘Zarb-e-Azb’” – (NGO Staff)*

#### Prioritizing of interventions

The study identified that there was no uniform, formal mechanism for prioritization of RMNCAH&N services in conflict areas. Most of the decisions were made at the center (Islamabad) and after devolution of powers in 2011, at the provincial capital and these decisions were majorly influenced by development partners and the funding agencies. There was usually no need-assessment (baseline data) conducted and strategies were formed at the central level with little or no involvement of the stakeholders at the peripheral level. Few participants from the development sector and the NGOs though did mention that prioritization of interventions is through need based assessments and desk-based literature reviews.

*“UN has conducted need based assessments and they have prioritized few areas in collaboration with the government. Currently, we are doing a project in which we have observed the need of health worker trainings and we are focusing on it … ” – (Academia_Professor)*

The priority of health service delivery had been on RMNCH&N services with a specific focus on immunization (specifically polio) and emergency obstetric care including essential newborn care. The greater emphasis on polio also have had a detrimental effect on the routine immunization program, although the focus of routine childhood immunization had increased in recent years. World Health Organization (WHO) had been leading the polio initiatives, supported by the government, NGOs and the security agencies. FATA is still unable to eradicate polio due to the porous border with Afghanistan, poor performance of lady health workers (LHWs) and resistance from the community, as there were many misconceptions among the residents despite of various education programs and food supplies provision to prevent refusals.

*“Polio eradication is the top most priority for Pakistan (including FATA). Second is basic EmOC services and essential new born care and nutrition services. There is less focus on communicable diseases, though the next third world war will be on communicable diseases” – (UN_Official)*

Among nutrition interventions, malnutrition screening and treatment had been prioritized through provision of ready-to-use therapeutic food (RUTFs) and food rations through outpatient services and referring the complicated cases to facilities or stabilization centers. IYCF and micronutrient supplementation have also been the areas of focus with an aim to avert immediate morbidity and mortality affecting vulnerable groups such as women and children and displaced populations. There have been no specific focus and programs for family planning and preventing early marriages except for an NGO working on it independently. The emphasis on increasing awareness and practices for breastfeeding was lacking and it was suggested to initiate programs solely focusing on improving IYCF practices in order to prevent undernutrition and reduce childhood morbidity and mortality. 

Despite the focus on RMNCAH, the coverage of essential interventions including ANC, SBA, IYCF is far from optimal. The rates of SBA and facility delivery are low, as most women preferred traditional birth attendants and apart from the socio-cultural reasons, the other reasons included far-away facilities and compromised quality of care in the healthcare facilities. There have been multiple interventions on conditional cash transfers (CCTs) in FATA to encourage women education, midwives training and educating women to seek healthcare and to improve nutritional and hygiene status, but the impact of these has not been formally assessed. People living in these areas have poor hygiene practices including lack of hand washing practices which contributes to disease.

The integration of mental health services and non-communicable services with the existing health delivery at the primary and secondary level was identified as a core need though it was not given a priority. This was due to limited resources and enhanced focus on managing malnutrition and communicable diseases from donors, development partners and government. Adolescent health was also another area which was largely ignored and no adolescent-specific services were prioritized.

#### Socio-cultural context

Destruction of the infrastructure, economic crisis, poverty, illiteracy, food insecurity, poor communication and cultural norms had contributed to increased malnutrition, maternal and child morbidity and mortality. The difficult terrain, sparse population, compromised road network and communication system imposes an even greater challenge for access in these conflict areas. Availability of emergency transport was also identified as an issue and contributed to mortality especially in emergency obstetric situations. Poverty and illiteracy dominates these communities and makes them vulnerable to the extremists groups and forces parents to let their children join the extremists groups to earn a living.

*“Why would a child do suicide bombing? It’s because he has a family with no money to eat or live, so family sacrifices one child. We should immediately respond to any anti Pakistan agenda and support positivity.” – (Government official)*

Cultural norms and practices has affected the accessibility of healthcare for women and children. These norms restrict women from travelling or leaving her house alone and it is considered a dishonor, if an adolescent was caught visiting a healthcare facility. The female was either restricted by males or by their mother in laws to visit a healthcare facility for ANC or PNC. Women have been groomed so that male can control and physically abuse them and women usually have no right or support to raise any concern.

*“I observed a case of twins; one was a female and other a male child. The female child wasn’t given much importance, as she was not breast fed by her mother and she was not vaccinated. Also, women lack decision making power here because it’s a male dominant society.” - (NGO_Official)*

#### Health workforce

One of the major bottlenecks in delivering health services was the non-availability of health professionals of all cadres and especially female health workers. There have been incidents of harassment, target killing, security threats and kidnapping of the health workforce specifically the polio workers. There have also been instances of land mines explosions, snatching of private vehicles of healthcare workers, hence they were reluctant to travel in their own vehicles and travelling in government vehicles made them prone to attacks from anti-state elements. The other major reasons apart from security issues included lack of housing facilities, unavailability of electricity, water, food and other basic necessities; hence it was difficult to attract workers even with higher salaries to work in these areas. Hiring outsiders is an issue, as they were reluctant to serve these areas and also have socio-cultural and language issues leading to reduced acceptability from the community. The political interference also affected health workforce availability, as people hired on political influence were less qualified, irregular on duties and lacked experience. The experienced and senior grade staff moved to major cities and the reliance in these rural areas was on junior staff. One strategy adopted in district Keich was to send senior staff on rotation basis so they were also based at the district center and had to go to far-flung areas for a week in a month and this was proving to be a successful model.

*“It’s like that there is no law and regulation in FATA, so majority of the appointments are due to political influence.” – (UN_Official)*

*“There is no security, no environment to work, no place to live and our health facilities are in pathetic condition. Why would anyone work here? I even got a threat of being kidnapped.” – (Govt. Official)*

During periods of intense conflict; community health workers if present, were the only available service providers for maternal and child health and even they were not providing services due to lack of monitoring, low salary package, absence of transport, difficult terrain and scattered population. There were no routine trainings for the health staff, but stakeholders did ensure trainings of LHWs on essential maternal and child care. Field staff were also trained on management of emergency situations such as natural disaster and epidemics, but there are not enough trainings for the health facility staff.

#### Service delivery

Most of the health facilities were primary health care facilities which were unable to fulfill all the health needs of the community and the available funding was not enough to ensure minimum infrastructure and equipment (water, electricity, beds, and supplies). The secondary care facilities were relatively well maintained but were few and the tertiary care facilities only existed in major cities. Many of the primary care healthcare facilities in FATA and few in the Makran division of Balochistan were not functional due to insecurity, political influence, unavailability of staff, damaged healthcare facilities and occupation of these facilities by the security agencies themselves. The stringent government regulations for NGOs to work in FATA and Balochistan, also impeded the work. There was also absence or lack of laboratory and radiology services and the facilities did not follow any Standard Operating Procedures (SOPs) or protocols although they were present. Staff absence especially female staff, capacity gap and compromised quality of care discouraged the community and this together with lack of regulations contributed to quacks and traditional and faith healers filling the void.

*“There are problems, 60% of our health facilities are functional and 40% are not functional and even those which are functional; need a lot of improvement.” - (UN_Official)*

#### Supplies and commodities

The availability and supply of essential medicine and commodities was a major obstacle and reasons for this included lengthy procurement procedures, inappropriate forecasting and inadequate distribution of supplies between facilities. The road blocks and curfews during the conflict further exacerbated the supply and hence compromised healthcare.

The slow and tedious procurement systems of government were mostly controlled centrally with few inputs from the district, hence often health facilities received medicines that were not required or demanded. Lack of funds and poor budget allocation were also a major reason for this poor management of supplies. The existing pharmacies were not up to the mark and medicines were kept in dust and sometimes were not been prescribed due to lack of professionals. The private pharmacies available in these areas also sold medicines and supplements of poor quality as no monitoring mechanism was in place. Stock-outs were also an issue sometimes when enrollments exceeded the targeted limit and secondly when there was a gap in supply at the end of the year. Sometimes the staff and the community also sold the nutritional supplements in the open market, which were provided to them by the government and the NGOs.

*“There is a large market, where you would find the supplies like nutritional supplements given to the community being openly sold.” – (NGO_Official)*

#### Monitoring and evaluation

The monitoring and evaluation section in the FATA Secretariat and Provincial Headquarters employ different methods of monitoring including household surveys, DHIS and LHW records. Specific surveys were also sometimes conducted by donors or organizations before, after and during the implementation of the projects. These systems though in place, were weak and the data collected was not regular and often of poor quality including fake or incomplete data, especially for DHIS. The reduced capacity and training of health staff in data collection and monitoring together with resistance to change behaviors and no specific budget for data collection posed additional challenges for adequate monitoring and evaluation. Even the data that was collected through various sources was also not effectively used to monitor progress or make informed decisions.

*“DHIS system is weak, the data entered is not correct. People are not trained for this and don’t even know where the data would be used.” – (Govt_Official)*

#### Finances

The budgeting and fund allocation is done through the central government for FATA and provincial government for Balochistan. Apart from government, major funds were received from Global Fund program, Gates foundation, GAVI, WHO, UNICEF amongst others. These available funds were insufficient to tackle the health needs of the population and a major barrier in successful implementation of the programs, while the transparent and efficient use of existing funds was another concern. There were also delays in release of funds and inadequate distribution between the districts and the health facilities. The capacity gap of the government at various levels also impeded the efficient utilization of funds and at times there were enough funds but remained underutilized. The specific programs in place operated and funded by various agencies also usually lacked sustainability mechanism and terminated abruptly when the funding from the donor subsided.

*“We have been implementing nutrition project since three years in FATA and we have not received complete funds from the donor.” – (NGO staff)*

#### Coordination

The study identified inter-organization coordination as an important factor in the successful roll-out of programs and uptake of interventions especially in conflict settings and cluster meetings were an important means of coordination. These cluster meetings though in place for various programs were conducted irregularly except for immunization and polio.

These cluster meetings were deemed useful in reducing duplication of effort and feedback provided in these meetings helped in improving the programs and their implementation. Usually these meeting were conducted after every 2 to 3 months but in the state of emergency, these meeting are conducted on daily and weekly basis. Cluster meetings for immunization were held monthly and led by WHO and were held at district and provincial level where the donors, government, district and field staff and program coordinators met and discussed progress and specific issues and deliberate on possible solutions. UNICEF usually led the nutrition cluster meetings but these meetings were rarely conducted. Infrequent meetings were also held between the provincial health directorate and the district staff.

*“Cluster meetings are important especially in an emergency situation. Our coordination team should be very strong so that it timely updates about any outbreak situation or severe situation. It is like an advocacy forum where needs are identified.” – (UN_Official)*

#### Natural disasters and emergencies

There was a disaster management authority present at the federal and provincial level and FATA to look after natural disasters. These were responsible for all the actions in emergency situations and also conducted trainings on safety measures to be taken during any disaster. They also had a nutrition plan for emergency situation.

There had been outbreaks of measles, pertussis, diphtheria and dengue in areas of FATA such as Bannu and Khyber agency, while measles was also reported in Makran Division of Balochistan. There was a Disease Surveillance and Response Unit which looked after these outbreaks, but usually the response was inadequate.

## Discussion

Pakistan has faced numerous and far reaching developments over the last few decades, including various conflicts and natural disasters which have immensely damaged the infrastructure and agriculture and have adversely impacted the overall economy and the achievement of many health related and other targets. There are gaps around the availability of data especially in the most sensitive areas of Pakistan, as we did not find any data from health facilities and household surveys for FATA and the facility based data from Balochistan was unreliable. Hence our findings were based on only qualitative data for FATA and both household quantitative and qualitative data for Balochistan.

The study suggests that BRDs are not the sole indicator to assess the severity of conflict. It is observed that areas which are occupied by anti-state elements don’t have BRDs, as they are in control of these forces and most of the deaths in conflict are due to the attacks of anti-state elements and they usually target major cities. As these major cities have better infrastructure, socio-demographics and health services, hence BRD only could not be a sole indicator to assess the severity of conflict and its impact on health. The quantitative analysis for Balochistan through a Delphi methodology shows that conflict does effect the delivery of RMNCAH&N services as the coverage of contraceptives, facility delivery, exclusive breast feeding, BCG, and care seeking for ARI was significantly lower for severe conflict districts when compared to minimal conflict districts. Other studies have also reported low coverage of SBA (38.8%), measles coverage (59.9%) in Pakistan [19] and less than 50% immunization coverage in 13 to 16 districts of Balochistan [20]. The lack in progress in RMNCAH&N indicators is also due to factors other than conflict and includes poverty, poor quality of care, urbanization, illiteracy among women, high rate of population growth, difficult geographic terrain in some areas and availability of trained staff only in urban centers [19, 21], but we believe that comparing the various districts of Balochistan amongst themselves adjusted to these other variances and hence we were able to assess the impact of conflict on the coverage of various interventions. Gender difference has also been a concern in Pakistan, however recent studies do not suggest significant differences by gender in healthcare utilization for both preventive and curative services, but the effect varies as women in urban area utilize healthcare more relative to the women living in rural areas [22–24].

The qualitative analysis highlights several factors affecting delivery of care in Balochistan and FATA which includes health workforce, service delivery, governance, security, health information system and resources (Table 5). Service delivery has been affected by planning and decision making at central level, lack of coordination, inadequate mobilization of funds, insecurity, lack of human resources due to reduced access and fear of attacks, difficult working conditions affecting quality of care, lack of supplies and equipment, non-functional healthcare facilities, reduced monitoring and supervision, reduced investments in all sectors and difficulties in procurement.

**Table 5:** Facilitators, barriers and recommendation affecting health system in conflict areas of Pakistan

	Facilitators	Barriers	Recommendations
Health Workforce	- Hiring of qualified local people along with incentives for retention	- Lack of female health workers	- Hire more female staff and reduce gender imbalance
		- Absenteeism and lack of capacity of healthcare staff	
		- Workers hired from outside face language and cultural issues	- Send female staff on rotation basis to conflict areas
		- Political influence and favoritism	- Hire local people and provide adequate training
		- Security threats	- Provide housing and basic necessities
		- Low salaries	- Merit based hiring
		- Absence of accommodation and basic facilities for doctors	- Doctors or staff to provide replacements when going on leave
		- Quacks are preferred by people over doctors	
Service Delivery	- Secondary facilities relatively well maintained	- Non-functional healthcare facilities	- SOPs should be implemented
	- Establishment of various new primary and secondary healthcare facilities	- Poor infrastructure	- Work on infrastructure for the uptake of health care intervention
		- Political influence	
	- Ambulatory service with staff care	- Quality of care compromised	- Stringent monitoring mechanisms using technology
	- Midwives and lady health workers visit homes	- Unavailability of transport for staff	
	- Service of institutional deliveries in presence of skilled birth attendant	- Changing demographic pattern	- Improve community awareness and mobilization activities
		- No arrangements for transport of complicated cases	
	- Social mobilization activities		- Improving LHWs functionality
Supplies and Commodities	- Different donors provide different supplies and services	- Curfews during the conflict blocked supplies to the facilities	- Procurement decisions at the district level
			- Procurement systems to simplified and made efficient
	- Enough supplies were provided	- Insufficient supply for commonly used drugs	
		- Delay in supplies from government	- Strict monitoring
		- Supplements sold in open market	
		- Allocation of budget for medicines not revised according to present needs	
		- Absence of diagnostic facilities	
Monitoring and Reporting	- Before and after surveys sometimes conducted	- Poor quality of data	- Promote E-Health
	- Internal monitoring was done	- No record of training or equipment distribution	- Improve quality of data
	- Third party monitoring on monthly basis for Polio	- Preference of manual work over computer use	- Do situational analysis before implementation
	- DHIS system for reporting	- Data not used for decision making	- Data to be used for decision
Finances	- Funding is done by donors and the government	- Delay in release of funds from the donors	- To ensure sustainability of funding for existing programs
Cluster meetings	- Seminars held for coordination	- Not regularly held for most programs	- Regular cluster meetings for all issues
	- Regular meetings held for Polio at district and provincial level		- Improve communication between center and district
Natural Disaster	- Disaster management authority present at provincial level		- Improve the functionality
	- Nutrition plan for emergency situations present		
Epidemics	- Disease Surveillance and Response Unit in FATA		- Proper forecasting and pre-emptive measures

LHW, Lady Health Workers, *SOPs* Standard Operating Procedure, *MnE* Monitoring and Evaluation, *DHIS* District Health Information System

The study suggests lack of data especially for FATA and the health facility data from DHIS was reported to be patchy and unreliable. In the absence of data, most of the decisions were made from the center (top-Down approach) rather than taking the bottom-up approach. Other studies highlights that despite of having a de-centralized system, Pakistan healthcare system faces a lot of problems due to lack of coherent and robust policies, poor information and monitoring system [23, 25]. Studies suggested inter-sectoral and cross-sectoral consultation for alignment of policies, programs and interventions [21, 25]. A study reported that health workforce face unemployment, lack of professional development and promotions due to which they are unable to fulfill their basic responsibilities [25]. To overcome the shortage of health work force, the currently vacant positions should be filled through identifying local people and regular trainings should be conducted to enhance professional development and motivation as have been suggested by other studies [26]. The current emphasis on polio program is due a multitude of factors and most importantly the global attention and funding to the polio program. Apart from polio, the next emphasis has been on screening and managing malnourished children with provision of food supplement, while the other maternal and child health programs though present but lack the required attention. There should be a greater emphasis on prevention strategies especially on breastfeeding, immunizations and water, sanittaion and hygiene. The stakeholders need to do a need prioritization exercise to decide on the priority context specific interventions and there is a need to go beyond communicable diseases and focus also on non-communicable diseases, mental health and addressing the health needs of adolescents.

The limitations of our study included reliance on secondary data for the quantitative analysis and lack of data from FATA. We also could not capture views of community in our qualitative study due to restricted access to conflict areas specifically FATA and measure their perceptions on the health services being provided and the quality of life and access to healthcare. Despite several limitations, our study presents important policy implications for the conflict areas of Pakistan. Pakistan should have specific strategies for areas affected by conflict, with multi-partisan support for sustainability. There should be a collative policy for health workers assuring trained and qualified professionals in urban and rural areas including capacity building of staff. For ensuring quality of health services, stringent mechanism should be in place for compliance with standard procedures especially at the health facility level. There is also a strong need of federal/district/regional coordination and public private partnership with a strong surveillance system to improve the health outcomes.

The health of the communities cannot be improved by focusing on the health sector alone, it requires a multi-sectoral approach including poverty alleviation, income generation, food security, infrastructure and communication, education, sanitation and hygiene. Working for peace is utmost important and ensuring security is inevitable to ensure optimal availability of health care services, thus pro-active steps should be taken to deliver the best health services possible in these conflict settings.

## Conclusion

Conflict has severely hampered the maternal and child health services in the conflict affected areas of Pakistan. Although the focus in health has been on RMNCAH&N, but a detailed plan of intervention prioritization, strategic planning and robust implementation has been lacking. There are multiple barriers in conflict contexts, although these cannot be mitigated to the fullest, but efforts should be made to negate these as much as possible. Improvements are possible with proper context-specific planning, good governance, adequate human resource allocation, efficient utilization of resources and stringent supervision and monitoring mechanisms.

## Data Availability

The datasets used for analysis in this study are available from the corresponding author on reasonable request.

## Abbreviations

ANC: Antenatal Care
ARI: Acute Respiratory Infection
BCG: Bacillus Calmette–Guérin
BRDs: Battle Related Deaths
CCI: Composite Coverage Index
CCT: Conditional Cash Transfer
DHIS: District Health Information System
DPT3: Diphtheria-Tetanus-Pertussis
Exclusive BF: Exclusive Breastfeeding
EmOC: Emergency Obstetric Care
FATA: Federally Administered Tribal Areas
FCS: Fragile Conflict Affected States
IDPs: Internally Displaced Persons
IYCF: Infant and Young Child Feeding
KP: Khyber Pakhtunkhwa
LHWs: Lady Health Worker
MDGs: Millennium Development Goals
NGO: Non-Governmental Organization
OPD: Outpatient Department
OR: Odds Ratio
ORT: Oral Rehydration Therapy
PDHS: Pakistan Demographic Health Survey
PNC: Postnatal Care
RMNCAH&N: Reproductive, Maternal, Newborn, Child, and Adolescent Health and Nutrition
RUTF: Ready to Use Therapeutic Food
SBA: Skilled Birth Attendant
SDGs: Sustainable Development Goals
SOPs: Standard Operating Procedures
UCDP: Uppsala Conflict Data Program
UN: United Nations
UNICEF: United Nations International Children’s Emergency Fund
WHO: World Health Organization
